# MINORITY & DIVERSE POPULATIONS I UNWAVERING STRENGTH IN THE FACE OF ADVERSITY: THE STRONG BLACK WOMAN, DEPRESSION, AND BEREAVEMENT

**DOI:** 10.1093/geroni/igz038.1896

**Published:** 2019-11-08

**Authors:** Danielle L McDuffie, Rebecca S Allen, Sheila Black, Martha R Crowther, Ryan Whitlow, Laura Acker

**Affiliations:** The University of Alabama, Tuscaloosa, Alabama, United States

## Abstract

This study sought to investigate grief outcomes among older African American (AA) women following the recent loss of a loved one. Whether or not the “strong Black woman” (SBW) schema of presenting unwavering strength despite adversity was present in recently bereaved older AA women, specifically related to depressive outcomes, also was explored. Eleven AA women aged 46 years and older (M=64.2), completed one time, in-person semi-structured interviews detailing their grief experiences. Interview transcripts were then coded by a team using an inductive qualitative approach. Four themes emerged throughout the women’s bereavement experience: 1) Acceptance of Loss and Preparation, 2) Coping as a Gradual Passing of Time, 3) Engaging in Other Activities to Cope with Loss, and 4) Helping Others Cope. About a third of the women in the sample reported being clinically depressed in accordance with the specifications for Major Depressive Disorder reported in the DSM 5. AA women in the sample were found to portray not only the stoicism consistent with the SBW schema, but also themes consistent with embodying the schema during their bereavement experience. More research and attention should be paid to AA women’s manifestations of depression, under the knowledge that AA women may have nontraditional depressive symptom presentations. Further, the tendency for the SBW schema to emerge during bereavement should be addressed with AA women in clinical practice, as lack of awareness of the use of this mechanism could lead to exacerbated, adverse adjustment.

